# Model-Assisted Guided-Wave-Based Approach for Disbond Detection and Size Estimation in Honeycomb Sandwich Composites

**DOI:** 10.3390/s21248183

**Published:** 2021-12-08

**Authors:** Piotr Fiborek, Paweł Kudela

**Affiliations:** Institute of Fluid Flow Machinery, Polish Academy of Sciences, 80-231 Gdańsk, Poland; imp@imp.gda.pl

**Keywords:** honeycomb sandwich structures, spectral element method, structural health monitoring, guided waves

## Abstract

One of the axioms of structural health monitoring states that the severity of damage assessment can only be done in a learning mode under the supervision of an expert. Therefore, a numerical analysis was conducted to gain knowledge regarding the influence of the damage size on the propagation of elastic waves in a honeycomb sandwich composite panel. Core-skin debonding was considered as damage. For this purpose, a panel was modelled taking into account the real geometry of the honeycomb core using the time-domain spectral element method and two-dimensional elements. The presented model was compared with the homogenized model of the honeycomb core and validated in the experimental investigation. The result of the parametric study is a function of the influence of damage on the amplitude and energy of propagating waves.

## 1. Introduction

Honeycomb Sandwich Composites (HSCs) are a type of multi-layered structure that are composed of the mid-core with the geometry of honeycomb sandwiched between thin skins. They are widely used in the aerospace, marine and automotive industries due to the high strength-to-weight ratio, high energy absorption capability and effective acoustic insulation. However, these complex structures are exposed to various types of damage that are not found in metal alloy materials, e.g., hidden disbonds between the skin and the core, delamination of the skin plates, or the core impact damage. They can occur either during a manufacturing process, storage or in-service life. Therefore, advanced methods are required for on-line damage detection.

The Guided Waves propagation method is a high-potential approach in SHM for damage detection in HSCs [1,2,3,4,5]. GW are mechanical waves that propagate in a bounded elastic medium, e.g., bars, beams, rods, plates and shells. An excitation and sensing of the GW can be realised by the lightweight and inexpensive piezoelectric transducers (PZT) [6]. The compact PZT can be surface-bonded to the inspected structure or even embedded between the composite plies so that the measurements can be conducted in situ.

Among numerous GW-based techniques developed for damage detection and localisation, the most popular are pitch–catch [7,8], pulse–echo [9,10], phase array [11,12] and time-reversal mirror [13,14]. For damage identification, some of them require a baseline to be determined. Due to the costs and time-consumption, experimental investigation is an inefficient approach to obtain references.

Lonkar et al. presented new model-assisted diagnostics for SHM [15]. The numerical model was used to determine the exact velocity of the wave propagating in the stiffened panel, which is essential for accurate damage identification. A combination of 3D scanning laser vibrometry measurements and the numerical model to reconstruct or update baseline signals for damage detection with guided waves was proposed by Aryan et al. [16].

Recently, model-based approaches have been developed to estimate the probability of detection for characterizing SHM techniques [17,18,19,20]. The most common numerical modelling of the phenomenon of GW in HSCs found in the literature is a calculation of the effective material properties of the honeycomb structure by the homogenisation process [2,21,22,23,24]. However, this method is not able to adequately represent the phenomenon of propagation of elastic waves in such material. A more accurate model will be achieved if the real geometry of the hexagonal cell is retained.

Ruzzenne et al. presented a parametric study to evaluate the dynamic behaviour of the honeycomb and cellular structures through the finite element model and the application of the theory of periodic structures [25]. Recently, the simulations of the wave propagation in HSCs have been conducted with commercially available finite element code [26,27,28,29].

However, the finite element method (FEM) modelling of GW is inefficient as it requires a significant amount of memory and is time-consuming. The computational efficiency of the FEM in case of GW modelling in HSCs can be improved by using the time-domain spectral element method (SEM). The SEM was originally used for the numerical solution of the fluid flow in a channel by Patera [30] but has also been successfully developed for elastic wave propagation [31].

Kudela proposed a model of the GW in HSCs by the parallel implementation of the SEM [32]. The wave excitation was realized by an external force applied at the point of the panel. However, this model had a large number (1.5 million) of degrees-of-freedom (DOFs), because cells of the core and skin plate were modelled by the three-dimensional (3D) spectral elements; however, the simulation was limited to only one skin plate and a small dimension of the HSC (179×159 mm).

The above-mentioned drawbacks were motivation to propose a new model of the HSC. In the present paper, the skin plates, adhesive layers and each wall of the hexagonal core were modelled by two-dimensional (2D) spectral elements. However, 2D elements have nodes only in a mid-plane; therefore, there is no direct linking between the two adjacent structures. This connection was implemented by interface elements based on Lagrange multipliers [33,34].

Additionally, the signal was generated and recorded with piezoelectric transducers (PZT). A non-matching interface between the transducers and the skin was used to avoid a too complex mesh—likewise to the interfaces developed for the FEM [35,36]. To the best of the authors’ knowledge, the present model has not been implemented yet for HSCs.

The parametric study conducted in the paper leads to the determination of a model-assisted damage identification function (MADIF), which defines the influence of the size of the composite defect on wave propagation. In this case, the defect is assumed to be a disbond between the skin and the core.

## 2. The Time-Domain Spectral Element Method Formulation

### 2.1. The Spectral Element Method

The general concept of the SEM is based on the idea of the FEM. The similarity of both methods lies in the fact that the modelled domain is divided into non-overlapping finite elements, and external forces and arbitrary boundary conditions are imposed in the particular nodes. The main difference between those methods is a choice of the shape function N=N(ξ), which is interpolated by a Lagrange polynomial that passes through the element nodes. The nodes are localized on the endpoint of an interval, ξ∈[−1,1], and the roots of the first derivative of Legendre polynomial *P* of degree p−1:

(1) $$\begin{array}{c} \left(1-\xi^{2}\right)P_{p-1}^{\prime }\left(\xi \right)=0. \end{array}$$

The approximation of an integral over the elements is achieved according to Gauss–Lobatto–Legendre (GLL) rule at points coinciding with the element nodes, and the weights w=w(ξ) calculated as:

(2) $$\begin{array}{c} w(\xi )=\frac{2}{p\left(p-1\right){\left(P_{p-1}\left(\xi \right)\right)}^{2}}. \end{array}$$

This approach guarantees a diagonal mass matrix. The shape functions and the weights for 2D or 3D elements are obtained by the Kronecker product of vectors of individual axes, denoted by ⊗ as follows:

(3) $$\begin{array}{cc} N(\xi ,\eta )=N(\xi )⊗N(\eta ), & N(\xi ,\eta ,\zeta )=N(\xi )⊗N(\eta )⊗N(\zeta ),\\ w(\xi ,\eta )=w(\xi )⊗w(\eta ), & w(\xi ,\eta ,\zeta )=w(\xi )⊗w(\eta )⊗w(\zeta ). \end{array}$$

### 2.2. 2D Spectral Modelling

According to the first-order shear deformation theory [37,38], the displacement field is expressed as:

(4) $$\left\{\begin{array}{c} u^{e}\left(\xi ,\eta \right)\\ v^{e}\left(\xi ,\eta \right)\\ w^{e}\left(\xi ,\eta \right) \end{array}\right\}=\left\{\begin{array}{c} u_{0}^{e}\left(\xi ,\eta \right)+z\varphi_{x}^{e}\left(\xi ,\eta \right)\\ v_{0}^{e}\left(\xi ,\eta \right)+z\varphi_{y}^{e}\left(\xi ,\eta \right)\\ w_{0}^{e}\left(\xi ,\eta \right) \end{array}\right\},$$

where $u_{0}^{e}$, $v_{0}^{e}$ and $w_{0}^{e}$ are nodal displacements, $\varphi_{x}^{e}$, $\varphi_{y}^{e}$ are the rotations of the normal to the mid-plane with respect to the axes *x* and *y*, respectively.

(5) $$\left\{\begin{array}{c} u_{0}^{e}\left(\xi ,\eta \right)\\ v_{0}^{e}\left(\xi ,\eta \right)\\ w_{0}^{e}\left(\xi ,\eta \right)\\ \varphi_{x}^{e}\left(\xi ,\eta \right)\\ \varphi_{y}^{e}\left(\xi ,\eta \right) \end{array}\right\}=N^{e}\left(\xi ,\eta \right){\hat{d}}^{e}=\sum_{n=1}^{q}\sum_{m=1}^{p}N_{m}^{e}\left(\xi \right)N_{n}^{e}\left(\eta \right)\left\{\begin{array}{c} {\hat{u}}_{0}^{e}\\ {\hat{v}}_{0}^{e}\\ {\hat{w}}_{0}^{e}\\ {\hat{\varphi }}_{x}^{e}\\ {\hat{\varphi }}_{y}^{e} \end{array}\right\}.$$

The nodal bending strain–displacement relations are given in the form:

(6) $$\epsilon_{b}^{e}=B_{b}^{e}{\hat{d}}^{e}=\left[\begin{array}{ccccc} \frac{\partial N^{e}}{\partial x} & 0 & 0 & 0 & 0\\ 0 & \frac{\partial N^{e}}{\partial y} & 0 & 0 & 0\\ \frac{\partial N^{e}}{\partial y} & \frac{\partial N^{e}}{\partial x} & 0 & 0 & 0\\ 0 & 0 & 0 & -\frac{\partial N^{e}}{\partial x} & 0\\ 0 & 0 & 0 & 0 & -\frac{\partial N^{e}}{\partial y}\\ 0 & 0 & 0 & -\frac{\partial N^{e}}{\partial y} & -\frac{\partial N^{e}}{\partial x} \end{array}\right]\left\{\begin{array}{c} {\hat{u}}_{0}^{e}\\ {\hat{v}}_{0}^{e}\\ {\hat{w}}_{0}^{e}\\ {\hat{\varphi }}_{x}^{e}\\ {\hat{\varphi }}_{y}^{e} \end{array}\right\}.$$

The nodal shear strain–displacement relations are given in the form:

(7) $$\epsilon_{s}^{e}=B_{s}^{e}{\hat{d}}^{e}=\left[\begin{array}{ccccc} 0 & 0 & \frac{\partial N^{e}}{\partial y} & -1 & 0\\ 0 & 0 & \frac{\partial N^{e}}{\partial y} & 0 & -1 \end{array}\right]\left\{\begin{array}{c} {\hat{u}}_{0}^{e}\\ {\hat{v}}_{0}^{e}\\ {\hat{w}}_{0}^{e}\\ \varphi_{x}^{e}\\ \varphi_{y}^{e} \end{array}\right\}.$$

### 2.3. 3D Model of the PZT Transducer

The displacement vector of the PZT transducer is composed of three translational displacements and is defined as:

(8) $$\left\{\begin{array}{c} u^{e}\left(\xi ,\eta ,\zeta \right)\\ v^{e}\left(\xi ,\eta ,\zeta \right)\\ w^{e}\left(\xi ,\eta ,\zeta \right) \end{array}\right\}=N^{e}\left(\xi ,\eta ,\zeta \right){\hat{d}}^{e}=\sum_{l=1}^{r}\sum_{n=1}^{q}\sum_{m=1}^{p}N_{m}^{e}\left(\xi \right)N_{n}^{e}\left(\eta \right)N_{l}^{e}\left(\zeta \right)\left\{\begin{array}{c} {\hat{u}}^{e}\left(\xi_{m},\eta_{n},\zeta_{l}\right)\\ {\hat{v}}^{e}\left(\xi_{m},\eta_{n},\zeta_{l}\right)\\ {\hat{w}}^{e}\left(\xi_{m},\eta_{n},\zeta_{l}\right) \end{array}\right\},$$

where ${\hat{u}}^{e}$, ${\hat{v}}^{e}$ and ${\hat{w}}^{e}$ are displacements of the element nodes in ξ,η and ζ direction.

The nodal strain–displacement relations are given as [39]:

(9) $$\epsilon^{e}=B_{d}^{e}{\hat{d}}^{e}=\left[\begin{array}{ccc} \frac{\partial N^{e}}{\partial x} & 0 & 0\\ 0 & \frac{\partial N^{e}}{\partial y} & 0\\ 0 & 0 & \frac{\partial N^{e}}{\partial z}\\ 0 & \frac{\partial N^{e}}{\partial z} & \frac{\partial N^{e}}{\partial y}\\ \frac{\partial N^{e}}{\partial z} & 0 & \frac{\partial N^{e}}{\partial x}\\ \frac{\partial N^{e}}{\partial y} & \frac{\partial N^{e}}{\partial x} & 0 \end{array}\right]\left\{\begin{array}{c} {\hat{u}}^{e}\\ {\hat{v}}^{e}\\ {\hat{w}}^{e} \end{array}\right\}.$$

The electromechanical coupling is governed by the linear constitutive equation of piezoelectric material according to [6,40], and this is defined as:

(10) $$\left[\begin{array}{c} \sigma \\ D \end{array}\right]=\left[\begin{array}{cc} c^{E} & -e^{T}\\ e & \epsilon^{S} \end{array}\right]\left[\begin{array}{c} S\\ E \end{array}\right],$$

where σ and S are the stress and strain components, respectively, $c^{E}$ is the stiffness coefficient matrix measured at zero electric field, **e** is the piezoelectric coupling tensor, $\epsilon^{S}$ is the electric permittivity, and **E** and **D** are the electric field and electric displacement measured at zero strain. The superscript T denotes a transpose matrix. The electric field is defined as:

(11) $$\begin{array}{c} E^{e}=-B_{\phi }^{e}{\hat{\phi }}^{e}=\left[\begin{array}{c} \frac{\partial N^{e}}{\partial \xi }\\ \frac{\partial N^{e}}{\partial \eta }\\ \frac{\partial N^{e}}{\partial \zeta } \end{array}\right]{\hat{\phi }}^{e}. \end{array}$$

where ${\hat{\phi }}^{e}$ is a nodal voltage of the transducer.

### 2.4. Displacements Coupling at the Substructures Interface

**Figure 1:**
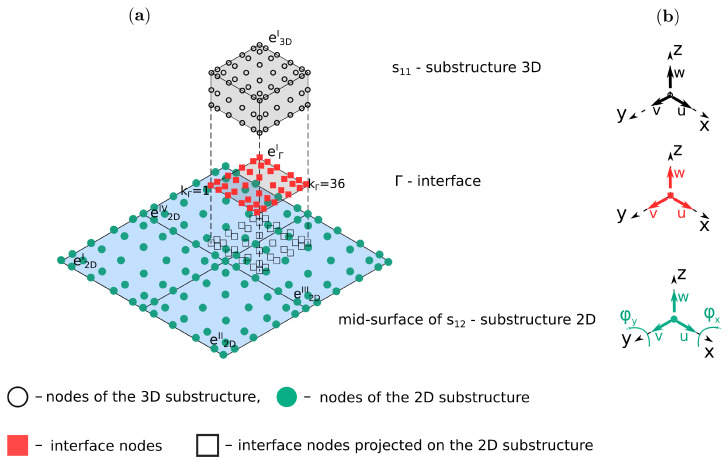
Non-matching interface setup: (**a**) interface coupling and (**b**) degrees-of-freedom of the interface and the substructures.

The present model of the sandwich panel consists of 2D and 3D elements. Moreover, there are non-matching grids between two adjacent substructures. These involve connecting them by imposing the compatibility of the displacements at the interface, see Figure 1. This type of connection is implemented through the interface elements based on Lagrange multipliers, which are interpreted as forces responsible for determining the appropriate displacements of nodes. The coupling can be expressed as:

(12) $${\left\{\begin{array}{c} u\\ v\\ w \end{array}\right\}}_{s_{i1}}^{\Gamma^{i}}-{\left\{\begin{array}{c} u\\ v\\ w \end{array}\right\}}_{s_{i2}}^{\Gamma^{i}}=\left\{\begin{array}{c} 0\\ 0\\ 0 \end{array}\right\},$$

where $s_{i1}$ and $s_{i2}$ are substructures connected by the interface $\Gamma^{i}$. For the whole structure, the Equation (12) can be written in the matrix form:

(13) $$\begin{array}{c} Gd=0, \end{array}$$

where **G** is the coupling matrix, which contains the equations to interpolate the substructures displacements at the interfaces, and d is a global displacement field for nS number of substructures, composed as:

(14) $$\begin{array}{c} d={\left\{\begin{array}{cccc} d_{1}, & d_{2}, & \ldots , & d_{nS} \end{array}\right\}}^{T}. \end{array}$$

The general formulation of the matrix **G** is presented in Algorithm A1 from Appendix B. The main task of the algorithm is to calculate shape functions for each adjacent substructures at the points $X_{p}=\left(x_{p}^{k},y_{p}^{k}\right)$, which are projections of the interface nodes onto these substructures.

The shape function can be calculated after finding an owner element and local coordinates of the points. The owner element is a spectral element in the domain of the substructure $s_{ij}$, which contains the interface node, for example, interface node $k_{\Gamma }=36$ (see Figure 1a) is located in the element $e_{3D}^{I}$ and $e_{2D}^{III}$ for the substructures $s_{11}$ and $s_{12}$, respectively. This can be found in two ways: using MATLAB’s built-in function inpolygon or more the efficient procedure proposed by Silva et al. [41], which was used in the current implementation.

The transformation from global to local coordinates was realised by the iterative method presented in the work of Li et al. [42]. The computational effectiveness of Algorithm A1 can be easily improved if certain precautions are taken. First, the mesh of the interface has to be based on the mesh from one of the substructures $s_{i1}$, $s_{i2}$, which may be referred to as a slave. Thus, the shape function takes only the values of one and zeros. Moreover, the code can be implemented in vectorized form rather than using for-loops.

### 2.5. Elementary Governing Equations of Motion

The classical equations of motion $M\ddot{d}+C\dot{d}+Kd=F$ known from FEM are complemented by piezoelectric and interface coupling. Thus, the governing equations are defined as:

(15) $$\begin{array}{c} M_{dd}\hat{\ddot{d}}+C_{dd}\hat{\dot{d}}+K_{dd}\hat{d}+K_{d\phi }\hat{\phi }=F-G^{T}\lambda , \end{array}$$

(16) $$\begin{array}{c} K_{\phi d}\hat{d}+K_{\phi \phi }\hat{\phi }=Q, \end{array}$$

where $M_{dd}$, $C_{dd}$ and $K_{dd}$ are the structural mass, damping and stiffness matrices, respectively; $K_{\phi d}={K_{d\phi }}^{T}$ are piezoelectric coupling matrices; $K_{\phi \phi }$ is the dielectric permittivity matrix, $\hat{d}$ is the vector of unknown nodal displacements, $\hat{\phi }$ is the electric potential vector, F is the nodal external force vector, Q is the nodal charge vector, λ is the Lagrange multiplier vector, and G is the interface coupling matrix $\left(\dot{\;}\right)=\frac{\partial }{\partial t}$. The formulae of the matrices are provided in Appendix A. The coupling is realised by imposing the traction forces as represented by a vector of Lagrange multipliers.

### 2.6. Parallel Implementation of the Internal Force Vector Calculation

The presented HSC model occupies much more operating memory than the homogenized one; thus, in order to achieve the solution in a reasonable time, the computation is performed using a multicore graphics processing unit (GPU). The most time-consuming operation in the Equation (15) is calculation of the internal force vector as: $F_{int}=K_{dd}\hat{d}$. It should be noted that the stiffness matrix $K_{dd}$ occupies a large amount of memory. Instead of allocating matrix $K_{dd}$, Kudela proposed a parallelized computation of the internal force vector [32].

The calculation is performed in three steps. First, the strain vectors are determined by multiplying the vectors of local node displacements and a sparse matrix containing local shape function derivatives. Then, the local internal force vector is obtained by multiplying the strain vectors by an appropriate material coefficient and a matrix of local shape function derivatives. Finally, the transformation of the local internal forces into the global forces is performed.

### 2.7. Transformation of the Core Elements

All core elements are rotated relative to both skins, and thus it is necessary to transform the degrees of freedom from the local coordinate system of the core to the global coordinate system. For this purpose, an additional sixth DOF is incorporated, i.e., rotation with respect to the *z*-axis:

(17) $$\begin{array}{c} {\hat{d}}_{g}^{e}={\left\{\begin{array}{cccccc} {\hat{u}}^{e} & {\hat{v}}^{e} & {\hat{w}}^{e} & {\hat{\varphi }}_{x}^{e} & {\hat{\varphi }}_{y}^{e} & {\hat{\varphi }}_{z}^{e} \end{array}\right\}}_{g}^{T}. \end{array}$$

First, the displacement vector is transformed from the global to local coordinate system by the direction cosines as follows:

(18) $${\hat{d}}_{l}^{e}={\left\{\begin{array}{c} {\hat{u}}^{e}\\ {\hat{v}}^{e}\\ {\hat{w}}^{e}\\ {\hat{\varphi }}_{x}^{e}\\ {\hat{\varphi }}_{y}^{e} \end{array}\right\}}_{l}={\left[\begin{array}{ccccc} V_{1}^{e}, & V_{2}^{e}, & V_{3}^{e}, & 0 & 0\\ 0 & 0 & 0 & V_{1}^{e}, & V_{2}^{e} \end{array}\right]}^{T}{\left\{\begin{array}{c} {\hat{u}}^{e}\\ {\hat{v}}^{e}\\ {\hat{w}}^{e}\\ {\hat{\varphi }}_{x}^{e}\\ {\hat{\varphi }}_{y}^{e}\\ {\hat{\varphi }}_{z}^{e} \end{array}\right\}}_{g},$$

where $V_{1}^{e}$,$V_{2}^{e}$ and $V_{3}^{e}$ are direction cosines of the core element. Then, internal forces are calculated according to guideline from Section 2.6 and transformed to a global coordinated system:

(19) $${\left\{F_{int}\right\}}_{g}^{e}=\left[\begin{array}{ccccc} V_{1}^{e}, & V_{2}^{e}, & V_{3}^{e}, & 0 & 0\\ 0 & 0 & 0 & V_{1}^{e}, & V_{2}^{e} \end{array}\right]{\left\{\begin{array}{c} F_{int}^{1}\\ F_{int}^{2}\\ F_{int}^{3}\\ F_{int}^{4}\\ F_{int}^{5} \end{array}\right\}}_{l}^{e}.$$

Additionally, a part of the mass matrix accounted for rotary inertia has to be transformed, and, in contrast to the internal forces vector, this has to be done only once in pre-processing as follows:

(20) $$J_{g}=\left[\begin{array}{ccc} {\left(J_{11}\right)}_{g} & {\left(J_{12}\right)}_{g} & {\left(J_{13}\right)}_{g}\\ & {\left(J_{22}\right)}_{g} & {\left(J_{23}\right)}_{g}\\ Sym. & & {\left(J_{33}\right)}_{g} \end{array}\right]={\left[\begin{array}{c} V_{1},V_{2},V_{3} \end{array}\right]}^{T}\;J_{l}\;\left[\begin{array}{c} V_{1},V_{2},V_{3} \end{array}\right].$$

As the matrix becomes non-diagonal after transformation, some approximation is necessary. Off-diagonal terms in the matrix given in Equation (20) are neglected following the analysis performed in [43].

### 2.8. A Solution of the Equation of Motion

Assuming b and f represent order lists of the electrode nodes and free nodes of the PZT, respectively, the electrical potential vector is rewritten:

(21) $$\hat{\phi }={\left\{\begin{array}{cc} \hat{\phi }\left(b\right) & \hat{\phi }\left(f\right) \end{array}\right\}}^{T}.$$

Then, Equation (16) is expressed as:

(22) $$\left[\begin{array}{c} K_{\phi d}\left(b,:\right)\\ K_{\phi d}\left(f,:\right) \end{array}\right]\left\{\hat{d}\right\}+\left[\begin{array}{cc} K_{\phi \phi }\left(b,b\right) & K_{\phi \phi }\left(b,f\right)\\ K_{\phi \phi }\left(f,b\right) & K_{\phi \phi }\left(f,f\right) \end{array}\right]\left\{\begin{array}{c} \hat{\phi }\left(b\right)\\ \hat{\phi }\left(f\right) \end{array}\right\}=\left\{\begin{array}{c} Q\\ 0 \end{array}\right\},$$

where the notation K(r,c) uses vectors r and c to extract rows and columns from the matrix K, respectively, and (:) means all rows or columns of K. The electrical potential of the free nodes can be extracted from Equation (22):

(23) $$\hat{\phi }\left(f\right)=-K_{\phi \phi }^{-1}\left(f,f\right)\left[K_{\phi d}\left(f,:\right)\hat{d}+K_{\phi \phi }\left(f,b\right)\hat{\phi }\left(b\right)\right].$$

Substituting Equations (21) and (23) into Equation (15), the equation of motion can be rearranged into the form:

(24) $$M_{dd}\hat{\ddot{d}}+C_{dd}\hat{\dot{d}}+\left(K_{dd}-K_{s}\right)\hat{d}=F+K_{a}\hat{\phi }\left(b\right)-G^{T}\lambda ,$$

where $K_{a}=K_{d\phi }\left(:,f\right)K_{\phi \phi }^{-1}\left(f,f\right)K_{\phi \phi }\left(f,b\right)-K_{d\phi }\left(:,b\right)$, $K_{s}=K_{d\phi }\left(:,f\right)K_{\phi \phi }^{-1}\left(f,f\right)K_{\phi d}\left(f,:\right)$. The unknown displacement vector ${\hat{d}}_{t}$ is found using a central difference algorithm [39]. Thus, Equation (24) is rewritten as:

(25) $$\begin{array}{c} \left(\frac{1}{\Delta t^{2}}M_{dd}+\frac{1}{2\Delta t}C_{dd}\right){\hat{d}}_{t+\Delta t}=F_{t}+K_{a}{\hat{\phi }}_{t}\left(b\right)-\left(K_{dd}-K_{s}\right){\hat{d}}_{t}+\\ +\frac{2}{\Delta t^{2}}M_{dd}{\hat{d}}_{t}-\left(\frac{1}{\Delta t^{2}}M_{dd}-\frac{1}{2\Delta t}C_{dd}\right){\hat{d}}_{t-\Delta t}-G^{T}\lambda_{t}, \end{array}$$

where Δt is the time increment.

Imposing the constrain Equation (13), the vector of Lagrange multipliers $\lambda_{t}$ can be extracted from Equation (25):

(26) $$\lambda_{t}={\left(GL_{+}^{-1}G^{T}\right)}^{-1}GL_{+}^{-1}[F_{t}+K_{a}{\hat{\phi }}_{t}\left(b\right)+\left.\left(\frac{2}{\Delta t^{2}}M_{dd}-K_{dd}+K_{s}\right){\hat{d}}_{t}-L_{-}{\hat{d}}_{t-\Delta t}\right],$$

where $L_{\pm }=\frac{1}{\Delta t^{2}}M_{dd}\pm \frac{1}{2\Delta t}C_{dd}$.

## 3. Experimental Validation

**Figure 2:**
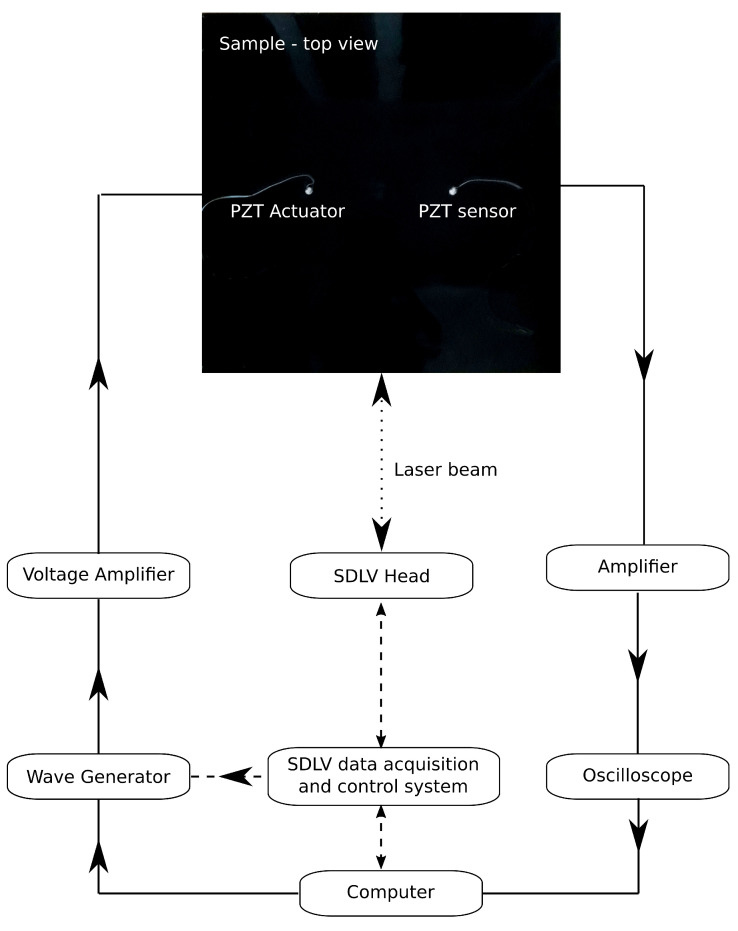
Experimental setup for the (1) SDLV measurement—dashed line and (2) PZT wave acquisition—solid line.

**Figure 3:**
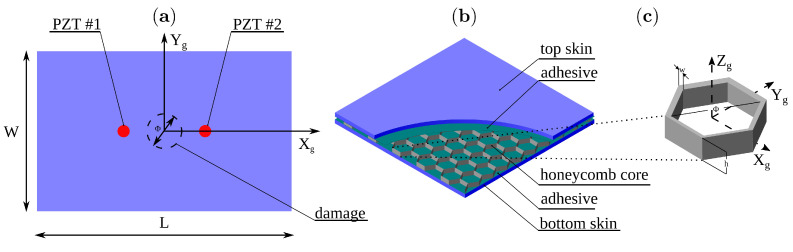
Sample configuration: (**a**) top view of the sample, (**b**) honeycomb sandwich substructures and (**c**) details of the honeycomb cell.

The presented model was validated with results from two experimental studies. The first one was performed for determination of the full wavefield of the propagating waves by the scanning laser Doppler vibrometer (SLDV, Polytec PSV–400). The second study was performed for wave acquisition by the PZT sensor. The schematic of the experimental setup is shown in Figure 2. The sample of interest was a not-regular hexagonal aluminium honeycomb bonded to one CFRP plate using the epoxy adhesive (Loctite EA3479B) as shown in Figure 3a).The subject of the parametric study was the effect of the disbond size on the propagating GW.

After a reference measurement was made on an intact sample, several measurements were taken for the subsequent damage introduced on the same specimen. The circular area of the core was detached from the adhesive at the centre of the plate using a sharp hooked tool. For this purpose, the bottom skin was omitted so that damage could be introduced. The damage size was controlled by its diameter $\Phi_{D}=\left[10,30,50,70,90,110,130\right]$ mm.

The generation and reception of elastic waves were achieved with a pair of PZT transducers mounted on the skin top surface with the cyanoacrylate glue. The coordinates of the actuator were $\left(x_{1},y_{1}\right)=\left(-100,0\right)$ mm, and for the sensor, $\left(x_{2},y_{2}\right)=\left(100,0\right)$ mm. The dimensions of the sample components were as follows:

- CFRP skin: L×W=500×500 mm, H=1.5 mm.
- Aluminium core: g=14.5 mm, w=0.1 mm, $h_{1}=11$ mm, $h_{2}=5$ mm, $l_{1}=10.4$ mm, $l_{2}=6$ mm.
- Epoxy adhesive: L×W=500×500 mm, H=0.3 mm.
- NCE51 PZT: $\Phi_{PZT}=10$ mm, h=0.5 mm.
- Cyanoacrylate glue: $\Phi_{CG}=10$ mm, h=0.05 mm.

The $N_{c}=5$ cycle Hann windowed signal at carrier frequencies $f_{c}=\left[75,100,125,150\right]$ kHz was generated using an arbitrary waveform generator (National Instruments, PXI 5413). The signal was amplified 40 times and supplied to the piezo actuator (Noliac, NCE51). Each measurement was conducted in the room temperature and averaged 20 times in order to improve the signal to noise ratio.

## 4. Numerical Simulations

### 4.1. Simulation Parameters

All structures used to create the sample were modelled in the simulation with the following elements: 2D for the core, epoxy adhesive and cyanoacrylate glue and 3D for the CFRP plate and PZT transducers. During the creation of the mesh, special attention was taken to reduce the number of non-zero values in the matrix G. While the inversion of the matrix $\left[{\mathrm{GL}}_{+}^{-1}G^{T}\right]$ is necessary to calculate the vector of Lagrange multipliers in Equation (26) and $L_{+}$ is a diagonal matrix, the sparsity of the matrix G has a significant effect on the computation cost.

**Figure 4:**
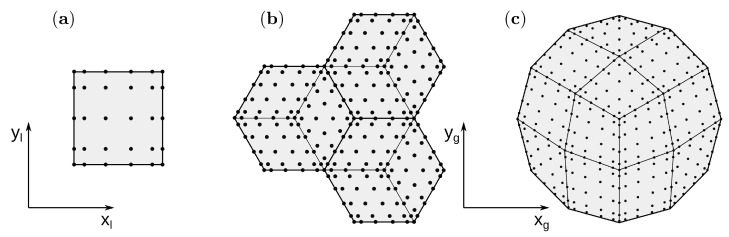
The mesh with the node distribution, (**a**) spectral element used for modeling the wall of the core, (**b**) excerpt of the skin plate and (**c**) cyanoacrylate glue mesh generated in GMSH.

One spectral element was intended for each wall of the honeycomb core, while the meshes of the skin plates and the adhesive layers were divided by three rhombus elements per area under the core cell. In this way, the interface nodes coincide with the nodes lying on the hexagon edges (thick line on Figure 4b). The mesh of the cyanoacrylate glue was generated using external software GMSH [44] (see Figure 4c) and joined to the plate by non-matching interface elements.

The PZT mesh coincides with the glue mesh. The convergence of the solution requires time increment to be less than a critical value, above which the displacements go to infinity. The critical value of time increment depends on the mesh size and the wave mode velocity. In the present model, convergence was achieved for 3 × 10^−9^ s. Additionally, the following number of nodes in the elements were used: the core 6×5, epoxy adhesive and cyanoacrylate glue 6×6, the plate 6×6×4 and PZT transducers 6×6×3.

**Figure 5:**
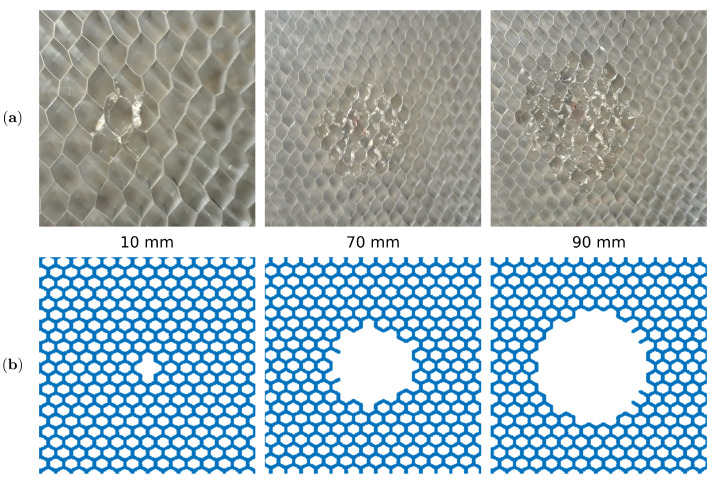
The damaged area in the: (**a**) experimental sample and (**b**) numerical mesh.

As the cells in the damaged area become distorted during core separation Figure 5a, the damage was modelled by removing the core elements in the disbond area as shown in Figure 5b.

The material properties used in the simulations are gathered in Appendix C.

### 4.2. Homogenized Model

For this paper, comparative studies were conducted between the current model and the homogenized one. In the simplified model, the values of the material constants of the panel core were calculated according to the method presented by Malek and Gibson [45]. The effective mechanical properties for an aluminium core are gathered in Table A2, while the properties for other structures, i.e., the skin, the epoxy adhesive, the cyanoacrylate glue, and the sensors remained unchanged. The core element has 6×6×4 nodes, and the mesh coincides with the plate mesh. The models of the other structures remain unchanged.

## 5. The Severity of Damage Estimation

**Figure 6:**
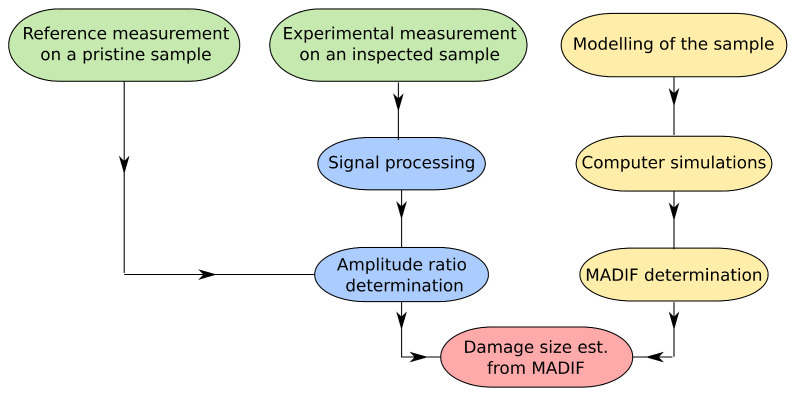
A flowchart representing the process for damage size estimation.

The severity of damage was estimated based on the function determined with the numerical simulation. A simple flowchart given in Figure 6 represents a process for the sample assessment. When the structure model is developed, several computer simulations for various damage sizes must be conducted to determine the MADIF.

**Figure 7:**
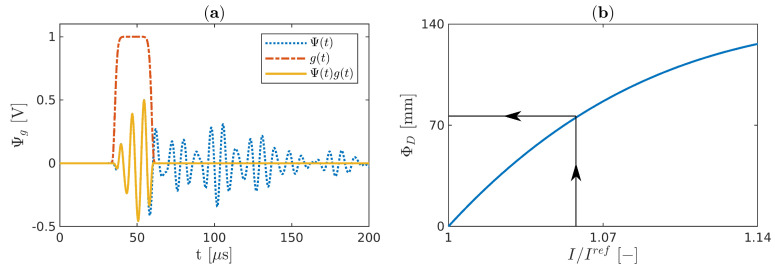
(**a**) The sensor signal Ψ(t) windowed by a flattened Gaussian window g(t) and (**b**) the damage size estimation from the MADIF.

The MADIF indicates the damage size according to measured damage index *I* normalized by the value obtained for the pristine sample $I^{ref}$. In the paper, two types of damage index *I* are considered: the energy $I_{eng}$ and the maximum value of the half-width of the first package arrived in the sensor $I_{amp}$, and these are defined as:

(27) $$\begin{array}{c} I_{eng}\left(\Phi_{D}\right)=\sum_{t=0}^{T}{\left(\Psi_{g}\left(t,\Phi_{D}\right)\right)}^{2},\;I_{eng}^{ref}=\sum_{t=0}^{T}{\left(\Psi_{g}\left(t,0\right)\right)}^{2}, \end{array}$$

(28) $$\begin{array}{c} I_{amp}\left(\Phi_{D}\right)=\max\left(\Psi_{g}\left(t,\Phi_{D}\right)\right),\;I_{amp}^{ref}=\max\left(\Psi_{g}\left(t,0\right)\right), \end{array}$$

where *T* is a period of the signal. $\Psi_{g}\left(t,\Phi_{D}\right)$ is for the damaged case scenario, whereas $\Psi_{g}\left(t,0\right)$ is for the pristine sample and it is realized in the same way by windowing the full-length signals of the sensor Ψ(t) with a flattened Gaussian window *g*(*t*) as follows:

(29) $$\begin{array}{c} \Psi_{g}\left(t\right)=\Psi \left(t\right)g\left(t\right)=\Psi \left(t\right)\exp\left(-{\left(\frac{t-t_{0}}{0.6005612w_{g}}\right)}^{12}\right), \end{array}$$

where $t_{0}$ is the centre and $w_{g}=0.5N_{c}/f_{c}$ is a half-width of the window. Windowing the signals ensures obtaining the signals without any reflections from the boundaries. The determination of $\Psi_{g}$ is pictured in Figure 7a.

In the time domain, an equivalent numerical signal to the signal registered by the PZT acquisition instrument is calculated as an average value of the electrical potential of the electrode surface

(30) $$\begin{array}{c} \Psi^{n}\left(t\right)=\frac{\int_{\Gamma_{e}}\phi d\Gamma }{\Gamma_{e}}, \end{array}$$

where n=1 and n=2 correspond to the homogenized and presented model, respectively.

The MADIF is achieved by approximating the inverse of the computed damage index that best matches the experimental one. Finally, the damage size $\Phi_{D}$ is obtained from the MADIF curve for measuring the normalized value of $I/I^{ref}$ as it is presented in Figure 7b.

## 6. Results

### 6.1. Comparison of the Models

**Figure 8:**
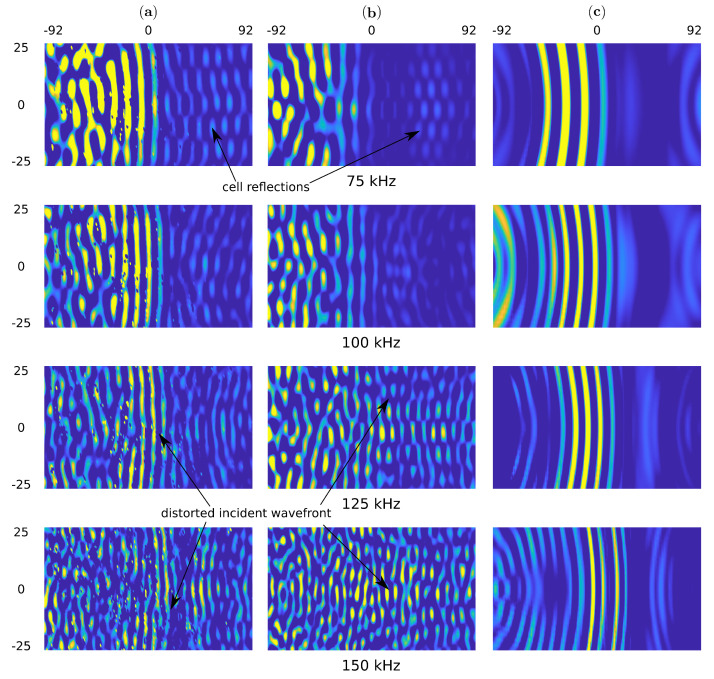
The top surface out of plane particle velocity snapshots in time 100 μs for (**a**) the experimental results obtained by using SLDV, (**b**) the present model and (**c**) the homogenized model in the pristine sample.

**Figure 9:**
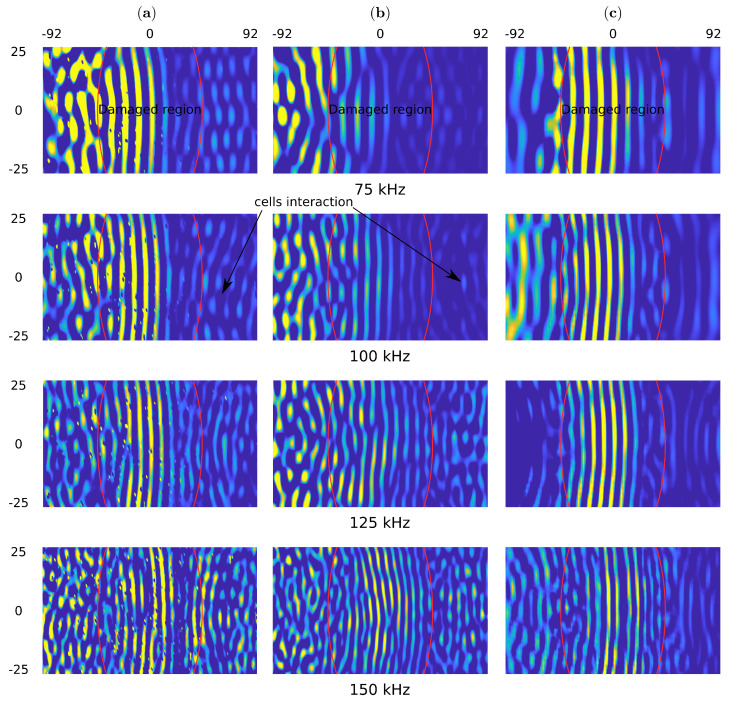
The top surface out of plane particle velocity snapshots in time 100 μs for (**a**) the experimental results obtained by using SLDV, (**b**) the present model and (**c**) the homogenized model in the sample with 90 mm damage.

The snapshots for the pristine and the damaged sample are shown in Figure 8 and Figure 9, respectively. One can observe the wave reflections in the core cells for experimental measurements and the present model. Additional, the front of the incident wave is distorted for the measurements of 125 and 150 kHz. The wavefront distortion in the present model is observed in the full range of frequency.

Such effects are not noticeable in the simplified model because the wave propagates smoothly through the structure. The wavefront improvement in the experiment and the present model is noticeable in the undamaged region and marked by the red curves in Figure 9. This is the effect of a lack of reflection with the core.

### 6.2. Model-Assisted Damage Identification Function

**Figure 10:**
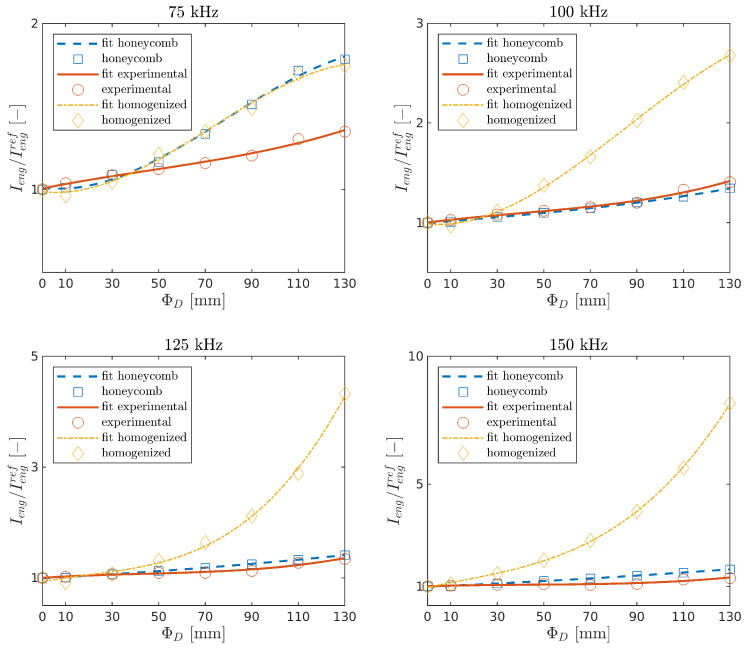
Relative change of the energy of the half of the first package in the function of damage size.

**Figure 11:**
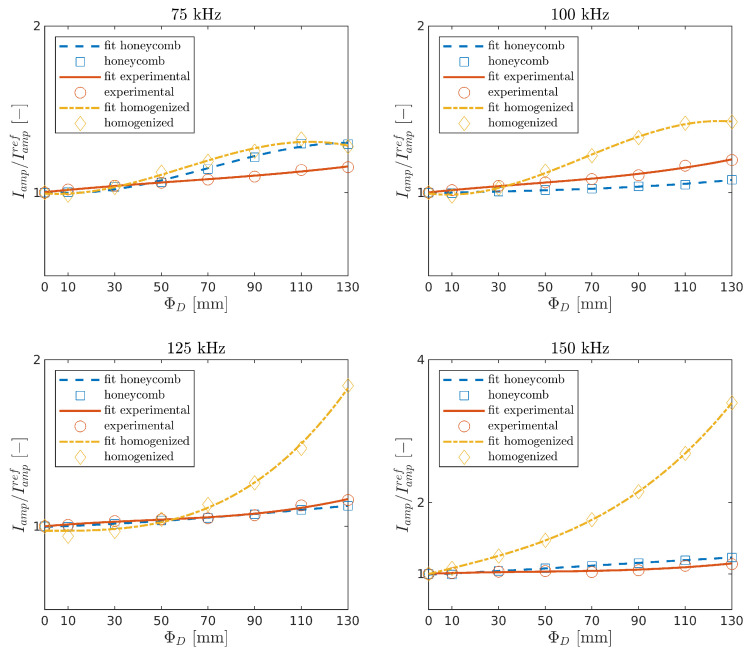
Relative change of the maximum amplitude of the first package in the function of damage size.

$I_{eng}$ and $I_{amp}$ were determined for experimental measurements and numerical calculations in the function of damage size and the carrier frequencies. The indices with the curve fitted by the polynomial interpolation of order three are shown in Figure 10 and Figure 11. It can be seen that both indices increase with the damage size in all cases. This is the effect of the leaky GW phenomenon [26].

Waves propagating through the plate lose energy in contact with the core. While GW propagates in the damaged area, such an effect does not occur, and thus the signal amplitude arriving at the sensor is higher. The present model is in good agreement with the experimental results for the tested frequencies $f_{c}=\left[75,100,125,150\right]$ kHz.

The homogenized model is in good agreement with the experiment for the lowest frequency, and the differences increase for higher frequencies. This issue may be related to the fact that the a shorter wavelength wave can lose more energy due to reflections at the edge of the damage of a homogenized model compared with a wave reflecting off the core cells.

To qualify the index as the MADIF, it must meet the condition of matching the numerical results with the experiment. The matching condition is that the value of the mean absolute error (MEA) must be less than an assumed threshold. The MEA is defined as:

(31) $$\begin{array}{c} MAE^{f_{c}}=\frac{\sum_{\Phi_{D}=0}\left|{\left\{I^{n}\left(\Phi_{D}\right)\right\}}^{f_{c}}-{\left\{I^{e}\left(\Phi_{D}\right)\right\}}^{f_{c}}\right|}{d}\times 100, \end{array}$$

where the superscripts *n* and *e* correspond to the numerical models and experimental measurements, respectively; and *d* is the number of damage cases. The threshold is the smallest to the most extensive damage ratio expressed as a percentage, i.e., $threshold=\Phi_{D}^{min}/\Phi_{D}^{max}\times 100$.

**Figure 12:**
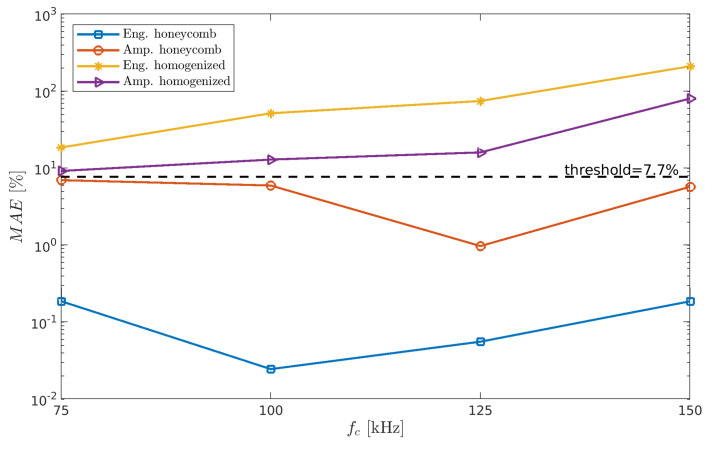
The mean absolute error of the indices.

**Figure 13:**
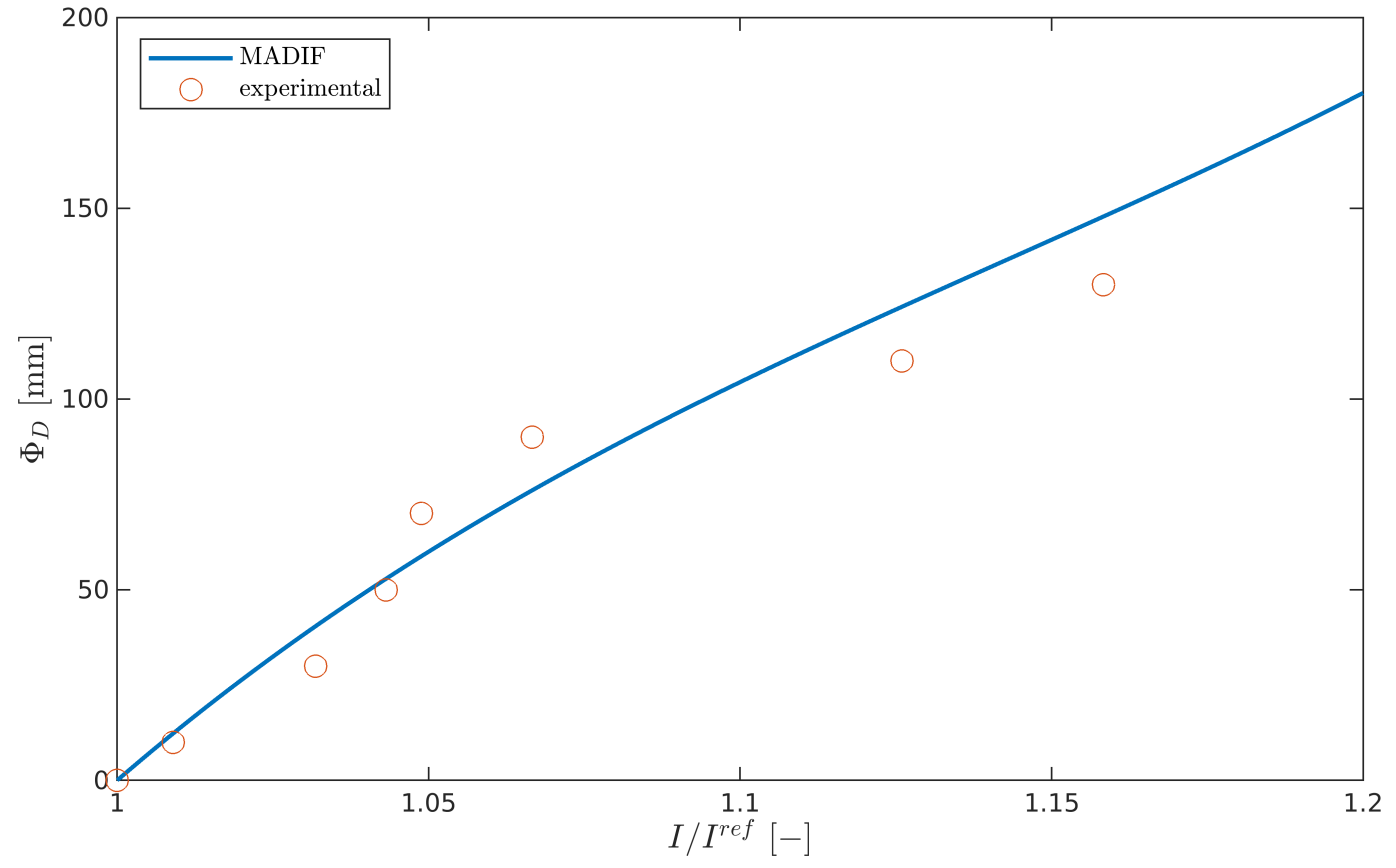
The model-assisted damage identification function (MADIF).

It can be seen from Figure 12 that the following indices satisfy the MAE condition: honeycomb index $I_{eng}$ for frequencies $f_{c}=\left[100,125\right]$ and $I_{amp}$ for $f_{c}=\left[75,100,125,150\right]$ kHz, but none from the homogenized model satisfy the MADIF selection criterion. In the case under consideration, the best fitting index turns out to be honeycomb $I_{amp}$ in 125 kHz; therefore, it was chosen for the MADIF, which is shown in Figure 13.

## 7. Conclusions

This paper presents preliminary research on the possibility of using a model-assisted approach to identify the severity of damage in a composite structure using GW propagation. For this purpose, the HSC model was implemented with the actual geometry of the honeycomb core. In contrast to full-structure homogenization, which is the most common HSC model found in the literature, the interactions of the propagating wave with the core cell walls were visible in the current model. The MADIF determined by the present model was in better agreement with the experimental measurements compared with the homogenized one.

In future works, our model will be used for parametric investigation to determine the MADIF in varied environmental conditions. The extended model will be usable in developing SHM scenarios.

## Data Availability

The data presented in this study are available on request from the corresponding author. The data are not publicly available due to due to privacy restrictions of the ongoing research.

## Appendix A

The formulae of matrices for 3D elements are:

(A1) $$\begin{array}{ccc} M_{dd}^{e} & = & \int_{V_{e}}N^{T}\rho NdV_{e}, \end{array}$$

(A2) $$\begin{array}{ccc} K_{dd}^{e} & = & \int_{V_{e}}{B_{d}^{e}}^{T}cB_{d}^{e}dV_{e}, \end{array}$$

where **c** is the stiffness tensor, ρ is the mass density, and $V_{e}$ is the element volume.

In the case of the 2D elements, the matrices are defined as:

(A3) $$\begin{array}{ccc} M_{dd}^{e} & = & \left[\begin{array}{cc} M^{e} & 0\\ 0 & J^{e} \end{array}\right]=\int_{\Omega_{e}}N^{T}\rho \left[\begin{array}{ccccc} h & 0 & 0 & 0 & 0\\ & h & 0 & 0 & 0\\ & & h & 0 & 0\\ & & & \frac{h^{3}}{12} & 0\\ Sym. & & & & \frac{h^{3}}{12} \end{array}\right]Nd\Omega_{e}, \end{array}$$

(A4) $$\begin{array}{ccc} K_{dd}^{e} & = & \int_{\Omega_{e}}{B_{b}^{e}}^{T}\left[\begin{array}{cc} A & B\\ B & D \end{array}\right]B_{b}^{e}d\Omega_{e}+\int_{\Omega_{e}}{B_{s}^{e}}^{T}\hat{A}B_{s}^{e}d\Omega_{e}, \end{array}$$

where $h=h_{t}+h_{b}$ is the element thickness, while $h_{t(b)}$ is the distance between mid-plane and top (bottom) surface of the element, and $\Omega_{e}$ is the element area:

(A5) $$\begin{array}{ccc} A & = & c_{ij}\;\left(h_{t}-h_{b}\right),\;i,j=1,2,6\\ B & = & 1/2\;c_{ij}\;\left(h_{t}^{2}-h_{b}^{2}\right),\;i,j=1,2,6\\ D & = & 1/3\;c_{ij}\;\left(h_{t}^{3}-h_{b}^{3}\right),\;i,j=1,2,6\\ \hat{A} & = & 5/4\;c_{ij}\;\left[h_{t}-h_{b}-4/3\left(h_{t}^{3}-h_{b}^{3}\right)/h^{2}\right],\;i,j=4,5. \end{array}$$

The dielectric conductivity matrix $K_{\phi \phi }^{e}$ and piezoelectric coupling matrix $K_{u\phi }^{e}$ are defined:

(A6) $$\begin{array}{ccc} K_{d\phi }^{e} & = & \int_{V_{e}}{B_{d}^{e}}^{T}e^{T}B_{\phi }^{e}dV_{e}, \end{array}$$

(A7) $$\begin{array}{ccc} K_{\phi \phi }^{e} & = & -\int_{V_{e}}{B_{\phi }^{e}}^{T}{\epsilon^{S}}^{T}B_{\phi }^{e}dV_{e}. \end{array}$$

## Appendix B

**Algorithm A1:** Matrix G formulation

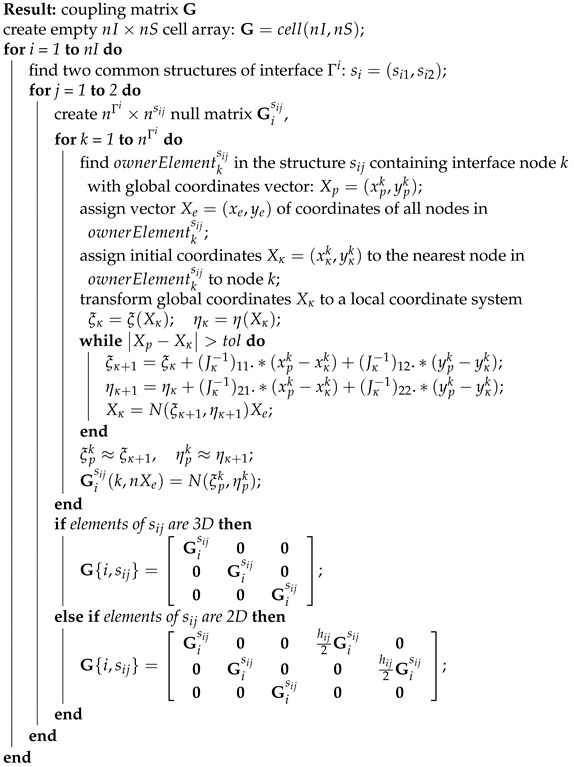

where nI and nS are the numbers of the interfaces and the structures, respectively; $n^{\Gamma^{i}}$ and $n^{s_{ij}}$ are the numbers of nodes of the interface *i* and the structure $s_{ij}$, respectively, $\eta_{p}^{k}$ and $\xi_{p}^{k}$ are local coordinates of $X_{p}$, respectively; $J_{\kappa }$ are Jacobians evaluated at $\left(\xi_{\kappa +1},\eta_{\kappa +1}\right)$; $N(\xi_{\kappa +1},\eta_{\kappa +1})$ is the shape function evaluated at $\left(\xi_{\kappa +1},\eta_{\kappa +1}\right)$; $nX_{e}$ is the vector of global order numbers of all nodes in the $Elements_{k}^{s_{ij}}$; $h_{ij}$ is the thickness of the structure $s_{ij}$; and tol is a termination criterion for iterations.

## Appendix C

The mechanical properties of the materials used in the simulations are gathered in Table A1, and effective elastic properties for a single layer of unidirectional CRFP are presented in Table A2.

**Table A1:** The mechanical properties of the materials.

Material	($E_{11}$)	($E_{33}$)	($\nu_{12}$)	(ρ)
	[GPa]	[GPa]	[–]	[kg/m${}^{3}$]
Carbon	275.6	27.6	0.2	1900
Epoxy	3.43	3.43	0.35	1250
Aluminium	71	71	0.33	2770
Epoxy adhesive	6	6	0.34	1200
Cyanoacrylate glue	3	3	0.34	1200

**Table A2:** The effective mechanical properties.

Material	$\left(E_{11}\right)$	$\left(E_{22}\right)$	$\left(E_{33}\right)$	$\left(G_{12}\right)$	$\left(G_{23}\right)$	$\left(\nu_{12}\right)$	$\left(\nu_{23}\right)$	(ρ)
	[GPa]	[GPa]	[GPa]	[GPa]	[GPa]	[–]	[–]	[kg/m${}^{3}$]
CFRP single layer	137	8.7	8.7	3.61	3.19	0.28	0.37	1569
aluminium honeycomb	40.0 × 10^−6^	40.0 × 10^−6^	663.2 × 10^−3^	24.0 × 10^−6^	148.0 × 10^−3^	0.998	0.02 × 10^−3^	25.36

The mechanical and piezoelectric properties of the PZT transducers are:

$$\begin{array}{c} c^{E}=\left[\begin{array}{cccccc} 134 & 88.9 & 90.9 & 0 & 0 & 0\\ 88.9 & 134 & 90.9 & 0 & 0 & 0\\ 90.9 & 90.9 & 121 & 0 & 0 & 0\\ 0 & 0 & 0 & 20.5 & 0 & 0\\ 0 & 0 & 0 & 0 & 20.5 & 0\\ 0 & 0 & 0 & 0 & 0 & 22.4 \end{array}\right]\left[\mathrm{GPa}\right] \end{array}$$

$$\begin{array}{c} e=\left[\begin{array}{cccccc} 0 & 0 & 0 & 0 & 13.7 & 0\\ 0 & 0 & 0 & 13.7 & 0 & 0\\ -6.06 & -6.06 & 17.2 & 0 & 0 & 0 \end{array}\right]\left[C\;m^{-2}\right] \end{array}$$

$$\begin{array}{c} \epsilon_{r}^{S}=\left[\begin{array}{ccc} 906 & 0 & 0\\ 0 & 906 & 0\\ 0 & 0 & 823 \end{array}\right]\left[-\right] \end{array}$$

$$\begin{array}{c} \rho =7850\;[\mathrm{kg}\;m^{-3}] \end{array}$$
